# Natural variation and domestication selection of *ZmSULTR3;4* is associated with maize lateral root length in response to salt stress

**DOI:** 10.3389/fpls.2022.992799

**Published:** 2022-10-26

**Authors:** Xiaomin Zhang, Tianze Zhu, Zhi Li, Zhongtao Jia, Yunyun Wang, Runxiao Liu, Mengling Yang, Qing-Bin Chen, Zhenjie Wang, Siyi Guo, Pengcheng Li

**Affiliations:** ^1^ Sanya Institute, Henan University, Sanya, Hainan, China; ^2^ State Key Laboratory of Crop Stress Adaptation and Improvement, School of Life Sciences, Henan University, Kaifeng, China; ^3^ Jiangsu Key Laboratory of Crop Genetics and Physiology/Key Laboratory of Plant Functional Genomics of the Ministry of Education/Jiangsu Key Laboratory of Crop Genomics and Molecular Breeding, Yangzhou University, Yangzhou, China; ^4^ Jiangsu Co-Innovation Center for Modern Production Technology of Grain Crops, Yangzhou University, Yangzhou, China; ^5^ Key Laboratory of Plant-Soil Interactions, Ministry of Education (MOE), College of Resources and Environmental Sciences, National Academy of Agriculture Green Development, China Agricultural University, Beijing, China

**Keywords:** maize (*Zea mays*), natural variation, salt stress, lateral root length, domestication selection, *ZmSULTR3;4*

## Abstract

Soil salinity is a major constraint that restricts crop productivity worldwide. Lateral roots (LRs) are important for water and nutrient acquisition, therefore understanding the genetic basis of natural variation in lateral root length (LRL) is of great agronomic relevance to improve salt tolerance in cultivated germplasms. Here, using a genome-wide association study, we showed that the genetic variation in *ZmSULTR3;4*, which encodes a plasma membrane-localized sulfate transporter, is associated with natural variation in maize LRL under salt stress. The transcript of *ZmSULTR3;4* was found preferentially in the epidermal and vascular tissues of root and increased by salt stress, supporting its essential role in the LR formation under salt stress. Further candidate gene association analysis showed that DNA polymorphisms in the promoter region differentiate the expression of *ZmSULTR3;4* among maize inbred lines that may contribute to the natural variation of LRL under salt stress. Nucleotide diversity and neutrality tests revealed that *ZmSULTR3;4* has undergone selection during maize domestication and improvement. Overall, our results revealed a regulatory role of *ZmSULTR3;4* in salt regulated LR growth and uncovered favorable alleles of *ZmSULTR3;4*, providing an important selection target for breeding salt-tolerant maize cultivar.

## Introduction

Maize (*Zea mays* L.) is a particularly important cereal crop planted worldwide, providing key resources for human food, animal feed and industrial products. Maize yield, however, is vulnerable to various environmental stresses. Among them, salt stress, which can be induced by excessive irrigation practices as well as factors related to climate change, could have a severe impact on corn production and has attracted increasing attention from researchers worldwide (Zhu, 2016; Ismail and Horie, 2017). A considerable number of studies have demonstrated that salt accumulation in soil solution not only provokes water deficit and nutrient imbalance, but also causes ionic toxicity to plant cells. This leads to plant metabolism disorder, impaired oxidation–reduction system, decreased photosynthesis, and ultimately suppressed plant growth and development (Yang and Guo, 2018; Van Zelm et al., 2020). In particular, severe salt stress occurring during the seedling stage is detrimental to developing seedlings (Sandhu et al., 2020; Chen et al., 2021). For instance, studies have shown that maize is hypersensitive to salinity stress (Farooq et al., 2015; Luo et al., 2019). Therefore, it is essential to identify the genetic variation contributing to maize salt tolerance in order to genetically improve the trait.

Previous studies have revealed that the exclusion of Na^+^ from the shoot is critical to the salt tolerance of crops. Allelic variation affecting the transcription of *HKT1*, *CIPK13* and *HAK4* has been reported to play a key role in sodium exclusion and, therefore, salinity tolerance (Rus et al., 2006; Munns et al., 2012; Roy et al., 2013; Zhang et al., 2019). Recently, *ZmSTL1*, a dirigent protein that confers variation in casparian strip thickness, was demonstrated to regulate transpiration-dependent Na^+^ exclusion, thereby improving the salt tolerance of maize plants (Wang et al., 2022). Another widely used proxy for salinity tolerance is the developmental plasticity of roots in response to salt stress (Julkowska et al., 2017; Li et al., 2021). Plant roots not only provide anchorage and participate in the uptake of water and mineral nutrients, but also act as the first organ sensing the salinity stress (Villordon et al., 2014; Wang et al., 2021). In the model plant Arabidopsis, the root system comprises a single embryonically formed primary root (PR) and several post-embryonically developed LRs of different orders. These traits describe the overall three-dimensional structure of root system, which enables plants to optimize resource acquisition and stress avoidance/escape (Julkowska et al., 2017; Jia and Von Wiren, 2020). LRs are specified in the pericycle cells of plants, with the exception in cereals that endodermal cells also contribute to LR formation (Van Norman et al., 2013; Yu et al., 2016). In general, LR development involves three developmental processes: LR primordium (LRP) initiation, LRP establishment and progression, and postemergence elongation (Casimiro et al., 2003). All these LR developmental checkpoints are highly susceptible to intrinsic and external environmental cues, such as nutrient availability and soil salinity (Karlova et al., 2021; Jia et al., 2022).

Maize establishes a typical fibrous root system composed of embryonic PR, seminal roots (SRs) as well as post-embryonic shoot-borne crown roots (CRs) and brace roots (BRs). All these root types undergo higher-order branching of LRs that greatly extend the exploration of soil volume (Bellini et al., 2014). Growth inhibition of maize root system as a whole by salt stress has been reported, although root-type specific plastic response has been observed (Zhang et al., 2015). Since lateral roots play an important role in water use efficiency, ion exclusion, and acquisition of water and macronutrients from soil, it is reasonable that changes in LR growth are of great adaptive value to plant survival under salt stress conditions (Faiyue et al., 2010; Ristova and Busch, 2014). Therefore, understanding the genetic components and exploring the natural variants that can enhance LR growth are of great importance for breeding stress-resilient crops.

In the present study, we showed by genome-wide association study (GWAS) that *ZmSULTR3;4* is associated with natural variation in LRL under salt stress condition. *ZmSULTR3;4* encodes a plasma membrane (PM)-localized sulfate transporter, with a preferential expression in the epidermal and vascular tissues of root. We further re-sequenced *ZmSULTR3:4* in 32 teosintes, 71 landraces, and 280 inbred lines, and a gene-based association analysis was conducted in inbred lines. The objectives of this study aimed to (1) identify natural variations in *ZmSULTR3:4* associated with LRL under salt stress condition; (2) examine the *ZmSULTR3:4* nucleotide diversity among teosintes, landraces, and inbred lines. (3) explore the role of *ZmSULTR3:4* in maize domestication and improvement. These findings may lay a foundation for further development of molecular markers for the genetic improvement of maize tolerance to soil salinilty.

## Materials and methods

### Plant materials and phenotypic evaluation

In this study, 32 teosintes, 71 landraces, and 280 maize inbred lines were used to evaluate LR length (LRL) at the seedling stage. LRL was collected with a paper roll system as previously described (Li et al., 2021). Briefly, seeds of uniform size were sterilized in 10% (v/v) H_2_O_2_ solution for 20 min following by washing with distilled water at least three times. They were then soaked in saturated CaSO_4_ for 6 h to synchronize germination, and further placed on moist filter paper for germination at 28°C and 80% relative humidity for 2 days in darkness. Afterwards, eight uniformly germinating seeds were transferred to the paper, which were vertically placed in a 39.5 × 29.5 × 22.5 cm black incubators containing 7.5 L Hoagland nutrient solution. The Hoagland nutrient solution were consisted of 2.0 mM Ca(NO_3_)_2_, 0.75 mM K_2_SO_4_, 0.65 mM MgSO_4_, 0.1 mM KCl, 0.25mM KH_2_PO_4_, 1 × 10^−3^ mM H_3_BO_3_, 1 × 10^−3^ mM MnSO_4_, 1 × 10^−4^ mM CuSO_4_, 1 × 10^−3^ mM ZnSO_4_, 5 × 10^− 6^ mM (NH_4_)_6_Mo_7_O_24_ and 0.1 mM Fe-EDTA. Five days after germination, the distilled water was replaced with Hoagland solution supplemented with or without 100 mmol·L^–1^ NaCl. The nutrient solution was refreshed every other day. Six individuals were harvested 14 days after germination. The roots were separated from the shoots and stored at 4°C. Then, the roots were scanned and analyzed using WinRHIZO software (Pro 2004b, Canada). Each treatment contained two independent biological replicates.

### GWAS and *ZmSULTR3;4*-candidate gene-based association mapping

A GWAS of maize LRL was performed by analyzing a maize natural population comprising 280 inbred lines (Li et al., 2019). LRL data were obtained from two replicated experiments. The association mapping panel was genotyped by genotyping-by-sequencing (GBS). After a quality control step (missing rate ≤ 20%), a total of 140,714 genomic single nucleotide polymorphisms (SNPs) were used for the GWAS with a minor allele frequency (MAF) ≥ 0.05, and their association with LRL was calculated by TASSEL 5.0 (Bradbury et al., 2007). The standard mixed linear model (MLM) was used, in which population structure (Q) and kinship (K) were estimated according to previous research (Bradbury et al., 2007; Liu et al., 2013). The genome-wide significant threshold was set as 1.96 × 10^-5^ (1/n, n represents the effective number of SNPs) as previously described (Li et al., 2021).

Genomic DNA was extracted from young leaves of teosintes, landraces, and inbred lines using the cetyltrimethylammonium bromide (CTAB) method. *ZmSULTR3;4*-based association mapping was performed with 280 maize inbred lines. The *ZmSULTR3;4* gene was resequenced using targeted sequence capture technology on the NimbleGen platform by Beijing Genomics Institute (BGI) Life Tech Co., China (Choi et al., 2009). The genomic sequence of *ZmSULTR3;4* (*GRMZM2G444801*) of the B73 inbred line was used as a reference for target sequence capture. Polymorphisms including SNPs and insertions/deletions (InDels) with a minor allele frequency (MAF) ≥ 0.05 were extracted and their associations with LRL was analyzed by TASSEL 5.0 using the standard MLM.

### Sequence analysis, genetic diversity analysis and neutral evolution test

MAFFT software was used to align the *ZmSULTR3;4* gene sequences, and BioEdit was used for manual improvement (Katoh and Standley, 2013). The gene features (5’-untranslated region (UTR), 3’-UTR, introns and exons) were demarcated according to the gene annotations of MaizeGDB (B73, AGPv3.31). DNASP 5.0 software was employed for sequence polymorphism analysis, genetic diversity analysis and neutral evolution test (Librado and Rozas, 2009). π and θ were used to evaluate the genetic diversity within individual population. The Tajima’s D as well as Fu and Li’s test were applied in the neutrality tests (Tajima and Genetics, 1989; Fu and Li, 1993).

### Identification of ZmSULTR proteins in the maize genome and phylogenetic analysis

Twenty-four sulfate transporter protein sequences from Arabidopsis and rice were used as references to search for ZmSULTR proteins in MaizeGDB (http://www.maizegdb.org/). The 12 SULTR proteins in Arabidopsis were downloaded from TAIR 10 (http://www.arabidopsis.org), and 12 SULTR proteins in rice were retrieved from the Phytozome database v10.0 (http://www.phytozome.net/eucalyptus.php). The full-length amino acid sequences of 32 SULTR members identified above were aligned with the Clustal X 1.83 software. Finally, the phylogenetic tree was constructed using the neighbor-joining method in MEGA-X with the default parameters.

### Expression profiling and RNA-seq analysis

The expression profiling of eight *ZmSULTR*s in various tissues were analyzed using the previously reported genome-wide gene expression profile of maize inbred line B73. A concise description of the tissues collected and sampling to create the gene atlas is presented in **Table 1** and **Table S1** of the published article (Sekhon et al., 2011). Expression data of 15 tissues from 60 developmental stages were collected and combined. The average normalized expression values of each gene in different tissues were obtained. For maize RNA-seq analysis, the uniform seeds of the maize inbred line B73 were selected and sterilized with 10% H_2_O_2_ for 30 min. The seeds were rinsed three times with distilled water to remove H_2_O_2_ from the surface of the seeds. The sterilized seeds were soaked in saturated CaSO_4_ for 6 h to promote germination and then placed on moist filter paper at 28°C and 80% relative humidity for two days. Eight germinated seeds were rolled up with brown germinating paper. The hydroponic experiment was conducted in an incubator at 28/22°C (day/night) with a relative humidity of 60% and a light intensity of 400 μmol.m^−2^.s^−1^. Hoagland solution was used instead of distilled water on day 3. At the same time, PEG 8000 solution (-0.8 MPa) was added to simulate soil drought stress, and the incubator was set at 40°C as the daytime temperature and 35°C for night to simulate a hot environment or 15°C as the daytime temperature and 10°C for night to simulate a cold environment. For the salt stress, the distilled water was replaced with Hoagland solution supplemented with 100 mmol·L^–1^ NaCl. Two independent biological replicates were set for each treatment. Tissues were collected from 30 roots and pooled for RNA-seq analysis. Total RNA was extracted using TRIzol reagent (Biotopped), and RNA integrity was assessed by a Bioanalyzer 2100 (Agilent). The 100-bp paired-end Illumina sequencing was performed at Berry Genomics (Beijing).

**Table 1:** List of parameters for the nucleotide polymorphism analysis of *ZmSULTR3;4*.

Parameters	Upstream	5'UTR	Coding region	3'UTR	Downstream	Entire region
Total length of amplicons (bp)	2252	255	5054	412	742	8715
Number of all of the sequence vanants	454	33	497	46	89	1119
Frequency of all of the sequence variants	0.202	0.129	0.098	0.112	0.120	0.128
Number of nucleotide substitutions (bp)	356	19	391	23	55	844
Frequency of polymorphic sites per bp	0.158	0.075	0.077	0.056	0.074	0.097
Number of InDels	98	14	106	23	34	275
Number of InDel sites	846	42	504	69	261	1722
Average InDel length	12.48	4.429	5.472	3.826	10.382	8.385
Frequency of indels per bp	0.044	0.055	0.021	0.056	0.046	0.032
π × 1000	17.83	1.76	7.02	8.34	5.65	9.15
θ × 1000	46.72	15.11	16.04	12.28	40.68	23.33
Tajima’s D	-1.893*	-2.240**	-1.723	-0.825	-2.466**	-1.874*
Fu and Li’s D *	-7.103**	-6.419**	-6.774**	-3.334**	-4.077**	-7.360**
Fu and Li’s F *	-5.049**	-5.7003**	-4.753**	-2.780*	-3.996**	-5.072**

*denotes statistical significance at the p < 0.05 level; ** denotes statistical significance at the p < 0.01 level.

For qPCR analysis of *ZmSULTR3;4*, root samples of A404 and A207 were collected after 24 h treatment with or without 100 mmol·L^–1^ NaCl. The growth procedure of plants were identical to phenotypic screening of LRL in natural population. Total RNA was isolated as mentioned above. cDNA was obtained using M-MLV Reverse Transcriptase, and qRT−PCR was performed using a SYBR Premix Ex Taq Kit (Takara) on a Step One System (Applied Biosystems, Shanghai, China). The 2^-ΔΔCt^ was used to calculate the relative expression of gene. Primer used for *ZmSULTR3;4* and *ZmUBI2* as internal control were listed in **Supplementary Table S1**.

### RNA *in situ* hybridization

Tissue embedding and RNA *in situ* hybridization were conducted as described by Gu et al., 2013. Briefly, the root tissues of 7-d-old hydroponically grown B73 plants were fixed in FAA for 12 h at 4°C. Then, the root tissues were embedded, and sectioned with a sliding slicer. The slides were dewaxed, digested with proteinase K (Roche), dehydrated with gradient ethanol, and hybridized by sense and antisense probes. After being washed, the slides were incubated with anti-digoxigenin-AP Fab fragments. Finally, the immunological detection was performed using the NBT/BCIP. The digoxigenin (DIG)-labeled RNA probes were listed in **Supplementary Table S1**.

### Subcellular localization and transient promoter activity assay of *ZmSULTR3;4*

The protoplast transient expression system were used for protein subcellular localization and promoter activity analysis. The coding sequence (CDS) of *ZmSULTR3;4* was cloned into *pGreenII-Ubi : GFP* vector by the *BamH* I site for protein subcellular localization. Protoplasts were isolated and collected from 14-day-old etiolated leaves of maize inbred lines (B73) as previously described (Yoo et al., 2007; Zhang et al., 2020). After a 14 h incubation, transformed protoplasts were counterstained with membrane dye FM4-64 (10 μM) for 1 min and then imaged with a confocal microscope (Leica TCS SP5).

For promoter activity assay, the ~1.3-kb and ~0.8-kb promoter fragments of *ZmSULTR3;4* were amplified from A404 and A207, respectively, and inserted in the upstream of *LUC* gene in the *pGreenII 0800-LUC* vector cleaved by the *Spe* I site. The transfection of protoplasts was conducted as above described. The Renilla luciferase (REN) driven by the 35S promoter was used as internal control to calculate the transfection efficiency. The detection of LUC signals were carried out as previously described (Chen et al., 2022). Four biological replicates were set up for each construct.

### Statistical analysis

The unpaired two-tailed Student’s *t* test was used to compare the differences in gene expression levels, LRL, and LUC acivity between the control and experimental groups. The Tajima’s D as well as Fu and Li’s test were applied to determine whether *ZmSULTR3:4* underwent selection. The data were considered different based on a threshold values of *p* < 0.05 and *p* < 0.01, as indicated by * and **, respectively.

## Results

### *ZmSULTR3;4* is associated with LRL under salt stress condition

**Figure 1:**
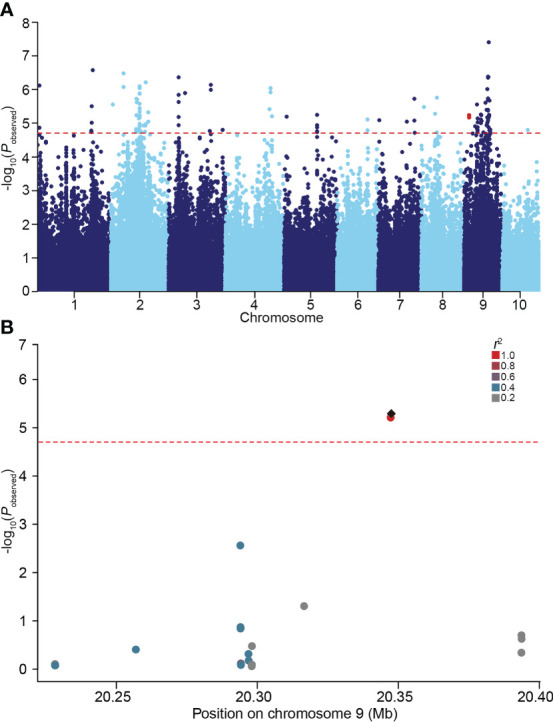
GWAS performed on the lateral root length (LRL) of maize under 100 mM NaCl condition. **(A)** Manhattan plot for the GWAS. The red dotted line represents the significance threshold (*P* = 1.96 × 10^−5^). Two SNPs located in *ZmSULTR3;4* were highlighted in red. **(B)** Local manhattan plot of the *ZmSULTR3;4* genomic region on chromosome 9. The 0.1-Mb genomic region on both sides of the most significant SNP was displayed. The most significant SNP was highlighted with a black diamond, while others were shown by dots and colored according to their LD (*r* ^2^) with the most significant SNP.

**Figure 2:**
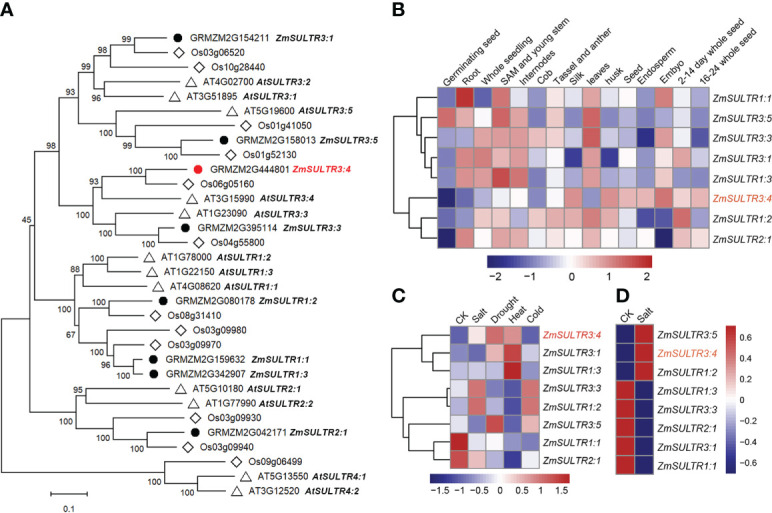
Phylogenetic and expression patterns of *ZmSULTR* genes in different types of tissue and stress response. **(A)** The phylogenetic tree of putative SULTR transporters in Arabidopsis, rice and maize. The neighbor-joining tree was constructed by MEGA-X with 1000 bootstrap values and the Poisson model. The scale denoteed the branch lengths. The gene identifiers and annotation were illustrated as black dots for maize, diamonds for rice, and triangles for Arabidopsis, respectively. **(B)** A heatmap showing the transcript levels of 8 *ZmSULTRs* in fifteen different tissues at various developmental stages. Normalized gene expression values were indicated in different colors. **(C)** Expression patterns of *ZmSULTR* genes in the leaves of 7-d-old hydroponically grown maize plants in response to salt, drought, heat, and cold stresses. The bar color represents the Z score of the FPKM of each gene under five treatments. **(D)** Expression patterns of *ZmSULTR* genes in the roots of 7-d-old hydroponically grown maize plants under salt stress conditions. The bar color for the Z score of the FPKM of each gene is shown on the right.

In our previous study, considerable phenotypic variation in LRL was revealed by analyzing a natural maize population under normal and salt stress conditions (Li et al., 2021). To further identify genes associated with variation in maize LRL, GWAS were performed on a natural maize population consisting of 280 inbred lines under normal and salt stress condition. Using a standard mixed linear model (MLM) incorporating population structure (Q) and kinship (K) as covariates, two SNPs within *GRMZM2G444801* on chromosome 9 were identified to be associated with LRL under salt stress condition, but not under normal condition (**Figure 1** and **Supplementary Figure S1**). *GRMZM2G444801* encodes a protein phylogenetically closely related AtSULTR3;4 in Arabidopsis (**Figure 2A**). The gene therefore was named *ZmSULTR3;4*. Notably, the expression of *ZmSULTR3;4* in roots was upregulated by salt treatment in six randomly selected inbred lines (**Supplementary Figure S2**), suggesting that *ZmSULTR3;4* may play a key role in plant salt tolerance.

### Expression patterns of *ZmSULTRs* and response to various stresses

Using Arabidopsis SULTR protein sequences as query to blast against maize database, eight putative *SULTR* genes were identified in the maize genome (**Figure 2A**). *SULTR* genes have been shown to play key roles in plant development, as well as in response to stress. To gain insights into the function of *ZmSULTR* genes, the tissue-specific expression patterns from the available transcriptomic data of maize B73 were evaluated. An expression heatmap of the eight *ZmSULTR* genes in different tissues at various developmental stages was constructed under normal growth condition. The results indicated that all *ZmSULTRs* showed a broad expression pattern in the tissues analyzed (**Figure 2B**). Additionally, genes clustered together showed similar expression patterns. Genes in class I were expressed at relatively high levels in the shoot apical meristem (SAM), young stems, internodes, tassels, anthers, and leaves but at low levels in endosperm and 16- to 24-day whole seeds. Genes in class II were expressed at relatively high levels in the silk, husk, seeds, and 2- to 14-day whole seeds but at low levels in the germinating seeds.

Transcriptome analysis was further conducted using maize seedling leaves to evaluate responses of *ZmSULTR* genes to different abiotic stresses, including salt, drought, heat, and cold. The results showed that *ZmSULTR3;4*, *ZmSULTR3;1* and *ZmSULTR1;3* were strongly induced by heat stress. *ZmSULTR3;3* and *ZmSULTR1;2* were induced by salt and cold, while they were downregulated by heat. In addition, *ZmSULTR3;5* was upregulated by drought and cold, but downregulated by salt and heat. Interestingly, the expression of *ZmSULTR1;1* and *ZmSULTR2;1* was downregulated by all types of stresses (**Figure 2C**). The expression of the eight *ZmSULTR* genes in response to salt stress was further examined in the roots of maize plants under salt stress condition (**Figure 2D**). Strong upregulation was found for *ZmSULTR1;2*, *ZmSULTR3;4*, and *ZmSULTR3;5* in the roots, hinting that these three genes may play a critical role in the root response to salt stress. In contrast, transcript level of *ZmSULTR1;1*/*1;3*/*2;1*/*3;1*/*3;3* were reduced upon salt treatment. Collectively, these results indicate that *ZmSULTRs* may play important roles in plant development and adaptation to different types of stresses.

**Figure 3:**
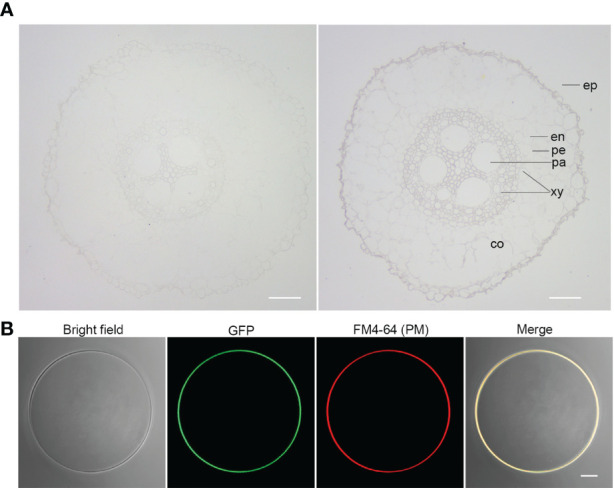
Tissue-specific expression and subcellular localization analysis of *ZmSULTR3;4*. **(A)** The tissue-specific expression of *ZmSULTR3:4* detected *via in situ* RNA hybridization in the roots of 7-d-old hydroponically grown maize seeldings. The tissue-specific expression of *ZmSULTR3;4* was detected using DIG-labeled RNA sense probes (left) and antisense probes (right). Ep, epidermis; en, endodermis; pe, pericycle; xy, xylem; pa, parenchyma; co, cortex. Bar = 100 μm. **(B)** ZmSULTR3:4 is localized exclusively in the plasma membrane (PM). The *Ubi : ZmSULTR3:4-GFP* expression cassette was transfected into maize B73 protoplasts. The transformed protoplasts were stained with FM4-64 stain for 1 min to marker the PM. Bar = 5 μm.

Given the strong association of *ZmSULTR3;4* with LRL under salt stress (**Figure 1**), we focus our study on *ZmSULTR3;4* in the present study. To gain insights into the function of *ZmSULTR3;4*, we then probe the tissue-specific expression of *ZmSULTR3;4* in roots of B73 using *in situ* mRNA hybridization. Results indicated that the sense probes of *ZmSULTR3;4* did not produce detectable hybridization signals (**Figure 3A**), while the strong signal of the antisense probe demonstated that *ZmSULTR3;4* was highly expressed in epidermal cells of the apical root zone and preferentially in the stele, especially in the pericycle cells and xylem parenchyma, but was absent in the cortex. Consistently, maize microarray data in the eFP browser confirmed that *ZmSULTR3;4* was highly expressed in the stele of roots and faintly expressed in the cortex (**Supplementary Figure 4**). We further transiently expressed *ZmSULTR3;4*-GFP fusion under the control of the strong constitutive *ZmUbi* promoter in maize leaf protoplasts to investigate the subcellular localization of *ZmSULTR3;4*. The Green fluorescence signal of ZmSULTR3;4-GFP overlapped well with the membrane marker FM4-64, indicating that ZmSULTR3;4 is a plasma membrane-bound protein (**Figure 3B**).

### Expression variation of *ZmSULTR3;4* associates with LRL under salt stress condition

**Figure 4:**
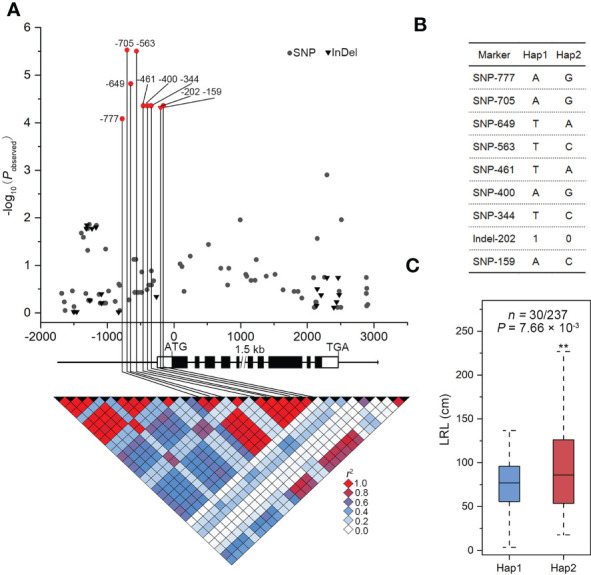
Genetic variation in *ZmSULTR3:4* is associated with the lateral root length (LRL) of maize seedlings under salt stress conditions. **(A)** Association analysis between the genetic variation of *ZmSULTR3:4* and the LRL of maize seedlings under salt stress. Black dots represent SNPs, and triangles denote InDels. The position of the start codon (ATG) is defined as ‘‘+1’’. The 5’- and 3’-UTRs and exons of *ZmSULTR3:4* are shown as open and filled boxes, respectively. The black lines represent gene promoters and introns. The *p value* is shown on a -log_10_ scale. The nine significant polymorphisms in the promoter are connected to the pairwise LD diagram with black vertical lines, illustrating that the nine variants are in strong LD (*r^2^* >0.8). **(B)** Significant markers of *ZmSULTR3:4* associated with LRL in different haplotypes. **(C)** The distribution of i LRL under salt stress conditions. n is the number of inbred lines belonging to each haplotype. Statistical significance was determined using a two-sided *t test*. "**”denotes statistical significance at the p < 0.01 level.

The association of SNPs in *ZmSULTR3;4* with LRL and transprtional upregulation of *ZmSULTR3;4* by salt stress promote us to further investigate how *ZmSULTR3;4* contributes to maize tolerance to salt. We therefore resequenced a 6.9-kb genomic fragment spanning the promoter to the 3’-UTR region of *ZmSULTR3;4* in 280 maize inbred lines. In total, 90 SNPs and 20 InDels (MAF ≥ 0.05) were detected (**Supplementary Table S2**). *ZmSULTR3;4* gene-based association analysis revealed nine polymorphisms (SNP-777, -705, -649, -563, -461, -400, -344, and -159 and InDel-202) in the promoter region were associated with LRL (*P* < 4.55 × 10^−4^). Among them, SNP-649 explained the most phenotypic variation (*r^2^* = 6.63%) in LRL. The nine DNA polymorphisms were in high LD (*r* ^2^ > 0.8), and divided the whole collection to two major haplotypes (Hap1 and Hap2) (**Figures 4A, B**). Statistically, the LRL of Hap2 inbred lines was on average longer than that of Hap1 under salt stress condition, suggesting that Hap1 is salt-sensitive, while Hap2 is salt-tolerant (**Figure 4C**).

**Figure 5:**
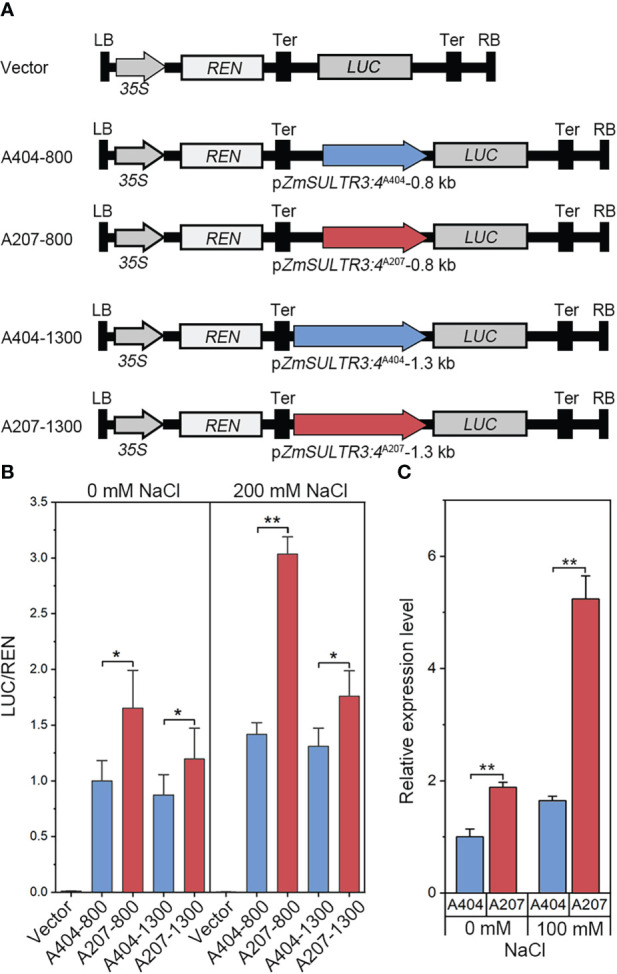
LUC enzyme activity driven by 0.8-kb and 1.3-kb promoter fragments of *ZmSULTR3:4* ^A404^ and *ZmSULTR3:4* ^A207^ under normal and salt stress conditions. **(A)** Vector diagram used to detect the effect of genetic variation of the promoter region on *ZmSULTR3:4* expression. A404-800, A207-800, A404-1300, and A207-1300 constructs harbor the promoter fragments of different *ZmSULTR3:4* alleles, including 800 bp from A404, 800 bp from A207, 1300 bp from A404, and 1300 bp from A207. **(B)** Transient expression assays of different promoter fragments from two. *35S:Renilla luciferase* was used as a positive control for transfection efficiency. Statistical significance was determined by a two-sided *t test*: *p < 0.05, **p < 0.01. **(C)** Relative expression of *ZmSULTR3:4* in roots of 14-d-old hydroponically grown A404 and A207 inbred lines under normal and 100 mM NaCl conditions.

Several significant genetic variations in the promoter region of *ZmSULTR3;4* suggests that expression variation of *ZmSULTR3;4* may be causal for the natural variation of LRL under salt stress. Therefore, *ZmSULTR3;4* expression was comprehensively analyzed in 167 maize inbred lines. The results indicated that the Hap2 inbred lines on average exhibits higher transcript level of *ZmSULTR3;4* than that of Hap1 inbred lines (**Supplementary Figure 3**), suggesting that differential expression of *ZmSULTR3;4* may contribute to the natural variation of LRL under salt stress. To further verify whether the genetic variation of promoter region contribute to the differential expression of *ZmSULTR3;4*, we assessed the promoter activity of two inbred lines contrasting in their sensitivity to salt stress using a transient expression system in maize protoplasts. Two promoter fragments (~0.8 kb or ~1.3 kb) of *ZmSULTR3;4* were cloned from the salt-sensitive inbred line A404 (Hap1) and the salt-tolerant inbred line A207 (Hap2) to drive the expression of luciferase (*LUC*) reporter gene and then were transformed into the maize protoplasts (**Figure 5A**). As shown by LUC expression, a stronger promoter activity was detected for *ZmSULTR3;4* ^A207^ than that of *ZmSULTR3;4* ^A404^ under both normal and salt stress conditions (**Figure 5B**). Furthermore, qRT−PCR analysis also indicated that the *ZmSULTR3;4* transcript level in A207 was higher than that in A404 under both normal and salt stress conditions (**Figure 5C**). Collectively, these data suggest that the natural genetic variation in the 0.8-kb promoter region differentiates the expression level of *ZmSULTR3;4*, which further conferred natural variation in maize salt tolerance.

### *ZmSULTR3;4* undergoes natural selection during maize domestication and improvement

**Figure 6:**
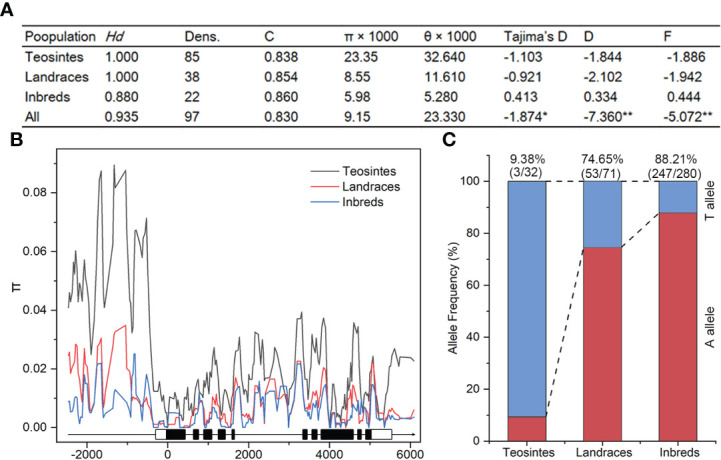
Analysis of nucleotide diversity (*π*) and allele frequency of *ZmSULTR3;4* in teosinte, landrace, and inbred lines. **(A)** Evaluation of nucleotide polymorphisms and neutrality tests of *ZmSULTR3;4*. *Hd* represents haplotype diversity; Dens. denotes the number of single nucleotide polymorphisms (SNPs) per 1000 bp; C represents sequence conservation; D and F represent Fu and Li’s D* and F*, respectively. Asterisks indicate statistical significance at the level of *p < 0.05, and **p < 0.01. **(B)** Nucleotide diversity (π) of teosinte, landrace, and inbred lines. The sliding window method was used to calculate π with a window size of 100 bp and a step size of 25 bp. A schematic illustrating the genomic region of *ZmSULTR3;4*, encompassing the upstream promoter region, introns (black lines), exons (filled boxes), and the 5’- and 3’-UTRs (open boxes). **(C)** Detection of allele frequency of SNP-705 in teosinte, landrace, and inbred lines. Number in the brackets indicated the count of accessions carrying the corresponding allele within respective population.

Nucleotide diversity reflects the historical process of maize domestication and artificial selection (Buckler and Thornsberry, 2002; Li et al., 2022; Yamasaki et al., 2007). To explore the evolutionary history of *ZmSULTR3;4*, we resequenced it in 32 teosintes, 71 maize landraces and 280 inbred lines. A 8715-bp genomic region was analyzed, including 2252-bp upstream, 255-bp 5′-UTR, 5054-bp coding region, 412-bp o 3′-UTR, and 742-bp downstream region, respectively (**Table 1**). A total of 1119 polymorphic sites were detected in all the varieties, comprising 844 SNPs and 275 InDels. The average frequencies of SNPs and InDels of the gene as a whole were 0.097 and 0.032, respectively. We observed that the upstream region and 3′-UTR showed the highest frequencies of SNPs and InDels, with a value of 1 per 6.33 bp and 1 per 17.86 bp, respectively. The nucleotide diversity analysis indicated that the overall nucleotide diversity (π × 1000) of *ZmSULTR3;4* was 9.15. Among the five investigated regions of *ZmSULTR3;4*, upstream region had the highest nucleotide diversity, with a π × 1000 value of 17.83, while the lowest π × 1000 value (1.76) was observed in the 5′-UTR (**Table 1**). We also compared the sequence conservation (C) and nucleotide diversity within three populations. The results show that the total C and π × 1000 values were 0.83 and 9.15, respectively (**Figure 6A**). Among three populations, landrace and inbred line exhibited higher conservation (C_L_ = 0.854 and C_I_ = 0.860) but lower nucleotide diversity (π × 1000_L_ = 8.55 and π × 1000_I_ = 5.98) than teosinte (C_T_ = 0.838 and π × 1000_T_ = 23.35). Furthermore, teosintes have higher nucleotide diversity across the whole gene regions compared with inbred lines and landraces, with the most significant divergence observed in the promoter region, suggesting higher selective pressure in the promoter region of *ZmSULTR3;4* during maize domestication (**Figure 6B**). Neutrality tests including Tajima’s D as well as Fu and Li’s tests were further applied to test whether *ZmSULTR3;4* underwent selection during maize domestication. We observed that Tajima’s D and Fu and Li’s values were not different within individual population, while Tajima’s D values of all test regions except for the coding region and 3′-UTR were significantly negative across three population. Likewise, Fu and Li’s values of all test regions were less than 0 (**Table 1**). We further analyzed the allele frequency of SNP-649 in teosintes, landraces, and inbred lines, considering that SNP-649 contributed the most to phenotypic variation. The frequency of salt-tolerant allele SNP-649^A^ was increased from 9.38% in teosinte to 74.65% and 88.21% in landrace and inbred lines, respectively (**Figure 6C**). These results collectively suggest that *ZmSULTR3;4* underwent purifying selection during maize domestication.

## Discussion

Soil salinity is a wide-spread constraint that limits plant growth and productivity. Therefore, it is of great significance to excavate important salt tolerance genes and elucidate their molecular regulatory mechanisms in salt tolerance. The root system, which is in direct contact with complex and changeable soil environment, is the first organ to encounter salt stress (Pierik and Testerink, 2014). Previous researches on the effect of salinity on root development showed that LR growth is more sensitive to salt than PR growth (Duan et al., 2013; Julkowska et al., 2017; Li et al., 2021). In maize, lateral roots account for most of the total root length (Lynch, 2013). Therefore, LRL can serve as an important trait for mapping salt tolerance QTLs and regulatory genes (Julkowska et al., 2017; Li et al., 2021).

Although many QTLs responsible for salt tolerance have been excavated (Cui et al., 2014; Zhang et al., 2018), the contribution of natural variation to phenotypic variations remains largely unknown. Numerous studies have shown that GWAS can successfully fine-map the QTLs that underlie complex quantitative traits in crops (Jia et al., 2019; Kuhlmann et al., 2020; Zhang et al., 2020). Maize is a kind of out-pollination crop, the genetic linkage disequilibrium (LD) decays very fast, and is estimated to be ≤ 2 kb in diverse inbred lines due to the high rate of recombination (Yan et al., 2009; Wang et al., 2016). In the present study, we performed LD-based GWAS of salt tolerance using 140,714 high-quality and dense SNP markers, with LD decreased to 0.2 at a distance between SNPs of approximately 50 kb, allowing for high-resolution QTL mapping (Li et al., 2021). we found that two SNPs in the *ZmSULTR3;4* gene was relevant to maize LRL under salt stress. When scanning the 0.1-Mb genomic region on both sides of the two SNPs, there were no other polymorphisms that are associated with maize LRL under salt stress. Therefore, it is plausible that *ZmSULTR3;4* is the only causal gene. Notably, the upregulated expression of *ZmSULTR3;4* by salt stress implies a positive role of *ZmSULTR3;4* in regulating LR growth and salt tolerance. Thus, the association of *ZmSULTR3:4* with salt tolerance seemed to be reliable, which might enhance our understanding of the genetic basis for maize salt tolerance.

Previous study also revealed that SULTRs in maize might play roles in adaptation to sulfur deficiency and adverse environmental conditions (Huang et al., 2018). In fact, several *SULTR3s* were identified to involve in in sulfate absorption and stress response. For instance, SULTR3;1 has been shown to localize in the chloroplast, and its loss decreases the sulfate uptake of the chloroplast (Chen et al., 2019). *SULTR3;5* was reported to play a role in root-to-shoot sulfate transport, with the mutation of *SULTR3;5* resulting in more sulfate accumulation in roots under low-sulfur conditions (Kataoka et al., 2004). While annotated as sulfate transporter, members in SULTR3 subfamily differs greatly in their substrate specificity. For example, the mutation of *OsSULTR3;3* reduces the concentions of sulfate and phosphate and modified the metabolic profile in rice grains (Zhao et al., 2016). Instead of transporting sulfate, vascular cambium-localized plasma membrane-bound *AtSPDT/AtSULTR3;4* and *OsSPDT* were demonstrated to transport Pi and mediate preferential distribution of phosphorus (P) to young tissues (Chen et al., 2019; Ding et al., 2020). *ZmSULTR3;4*, the closest ortholog of *AtSULTR3;4*, is also localized to the PM, suggesting there may be a conserved function in different plant species. The preferential expression of *ZmSULTR3;4* in the epidermal and vascular tissues of roots indicates that it may play an important role in transporting mineral elements to young tissues, allowing them to withstand malnutrition caused by high salt levels. Further research will be necessary to understand the *in planta* function of *ZmSULTR3;4*.

Then, we resequenced the *ZmSULTR3;4* gene to verify the results of association analysis and more accurately identify genetic variation related to maize salt tolerance. The results revealed nine significant genetic variants residing in the 0.8-kb promoter region of *ZmSULTR3;4*. Although nine significant natural variations were located in the *ZmSULTR3;4* promoter, the inbred lines mainly classified into two haplotypes. Compared with the inbred lines of Hap1, the inbred lines of Hap2 showed longer LRs under slat stress, suggesting that *ZmSULTR3;4* ^Hap2^ allele would be valuable in breeding program seeking to improve salt tolerance in maize germplasms. Consistent with the longer LR length in *ZmSULTR3;4* ^Hap2^ allele, we observed also higher *ZmSULTR3;4* expression in the *ZmSULTR3;4* ^Hap2^ inbred lines, suggesting that allelic variants in the promoter region may impact the transcript level of *ZmSULTR3;4* that confers different sensitivity to salt in maize population. Luciferase (LUC) and *β*-glucuronidase (GUS) are routinely used as reporters for the quantitative measurement of gene expression in transient expression using mesophyll protoplasts (Yoo et al., 2007; Tian et al., 2019). Supporting this conclusion, LUC activity driven by the 0.8-kb and 1.3-kb promoter fragments of *ZmSULTR3;4* ^Hap2^ was greater than that of *ZmSULTR3;4* ^Hap1^ promoter fragment, suggesting that functional allele resides in 0.8-kb promoter region. In fact, numerous studies have shown that regulatory polymorphisms upstream of genes as a major driver that differentiates gene expression and lead to changes in plant phenotypes (Salvi et al., 2007; Mao et al., 2015; Wang et al., 2016; Jia et al., 2020; 2021). For example, genetic variations in the promoter region of *ZmVPP1* and *ZmNAC111* regulate the gene expression, which is closely associated with variation of plant drought tolerance (Mao et al., 2015; Wang et al., 2016). An insertion upstream of *Vgt1* (*Vegetative to generative transition 1*) represses gene expression and affects maize flowering-time (Salvi et al., 2007). Unfortunately, the strong LD among these nine SNPs prohibits us to identify the causal functional allele associated with natural variation of LR length. In future precise nucleotide substitution of these nine variants will allow to fine-map the causal variant differentiating the expression of *ZmSULTR3;4.*

Genetic and archeological evidence has suggested that maize was domesticated from its wild ancestor, teosinte, in southwestern Mexico about 9000 years ago. The bottleneck effect of domestication resulted in a stark decrease in nucleotide diversity (Wang et al., 2017; Allaby et al., 2019). The rich genetic diversity is the basis of crop genetic improvement (Yan et al., 2011). In this research, nucleotide polymorphisms of *ZmSULTR3;4* were analyzed in teosintes, landraces, and inbred lines through resequencing. The genetic diversity of *ZmSULTR3:4* in teosintes, landraces and maize inbred lines decreased in turn, suggesting that approximately three-quarters of the genetic diversity in the *ZmSULTR3:4* genome was lost during the domestication process. Moreover, the apparent reduction of nucleotide polymorphisms in the promoter region of *ZmSULTR3:4* suggested that this region might have been subjected to greater selection pressure. The neutrality tests also revealed that *ZmSULTR3;4* might be selected during maize domestication and improvement. Indeed, the frequency of the favourable allele of *ZmSULTR3;4* increased gradually during the domestication from teosinte to landraces, and the improvement from landraces to maize inbred lines, which strongly reflects a breeding history involving selection. Similarly, a few yield- or stress-related genes, *e.g.*, *tb1*, *KRN4*, and *bZIP68*, have been reported undergone strong artificial selection during maize genetic improvement (Studer et al., 2011; Liu et al., 2015; Li et al., 2022). Although the salt-induced expression pattern of *ZmSULTR1:2* and *ZmSULTR3:5* was similar to that of Z*mSULTR3:4* in the roots, our GWAS analysis did not reveal an association between these two genes and the phenotype, which may be due to the limitation of population material or the choice of traits for salinity tolerance. New populations and phenotypic traits need to be developed to reveal the genetic architecture of maize salt stress response. Some important genes or loci responsible for salt mediated LR growth may also be missed in our GWAS due to SNP density and low frequency of some markers as well as the lacking of structure variations such as presence/absence variations (PAVs) and copy number variations (CNVs) in our genotype datesets. To our knowledge, *ZmSULTR3;4* was the first gene identified to play a regulatory role in salt-regulated LR growth and underwent domestication and improvement in maize. The biological function and regulatory network of *ZmSULTR3;4* need to be further investigated using approaches such as CRISPR-Cas9 and overexpression. Although some progress has been made in the study of plant SULTRs, the function of SULTRs in maize is still poorly understood. It is also unclear whether other *ZmSULTR* members were under selection during maize domestication and improvement. Further research is required to understand the biological function and selective characteristic of *ZmSULTRs*. Collectively, the identified natural variants and elite haplotype of *ZmSULTR3;4* may be used to improve maize root traits and salinity tolerance by molecular breeding.

## Data availability statement

The original contributions presented in the study are publicly available. This data can be found here: NCBI, PRJNA683126.

## Author contributions

XZ, PL, TZ, and ZL performed the experiments and drafted the manuscript. PL, ZJ, and SG conceived the experiment and revised the manuscript. XZ, MY, RL, ZW, and QC analyzed the data. All authors reviewed and approved this submission.

## Funding

This research was supported by the National Natural Science Foundation of China (32201726 and 31972487), the Open Project Funding of the State Key laboratory of Crop Stress Adaptation and Improvement, the Science and Technology Development Plan Project of Henan Province (212102110152 and 222102110006), the High-end Talent Project of Yangzhou University, Qing Lan Project of Jiangsu Province, and the Priority Academic Program Development of Jiangsu Higher Education Institutions (PAPD).

## Conflict of interest

The authors declare that the research was conducted in the absence of any commercial or financial relationships that could be construed as a potential conflict of interest.

## Publisher’s note

All claims expressed in this article are solely those of the authors and do not necessarily represent those of their affiliated organizations, or those of the publisher, the editors and the reviewers. Any product that may be evaluated in this article, or claim that may be made by its manufacturer, is not guaranteed or endorsed by the publisher.

## Supplementary material

Supplementary Figure 1 GWAS performed on lateral root length (LRL) under normal growth condition. The red horizontal dashed line depicted the significance threshold (*P* = 1.96 × 10^−5^).

Supplementary Figure 2 The relative expression level of *ZmSULTR3;4* in response to salt stress in roots of six hydroponically grown maize inbred lines. The data represented the mean of two replicates.

Supplementary Figure 3 Transcript level of *ZmSULTR3;4* in roots of different maize inbred lines under normal growth condition. Statistical significance was detected by a two-sided *t-test*; ***p* < 0.01.

Supplementary Figure 4 Relative expression of *ZmSULTR3;4* in the different developmental zones of primary roots. The data were retrieved from the Maize eFP Browser (http://bar.utoronto.ca/efp_maize/cgi-bin/efpWeb.cgi).
