# Supplementary figures and images for: Natural variation and domestication selection of ZmSULTR3;4 is associated with maize lateral root length in response to salt stress
Source: Front Plant Sci. 2022 Oct 26;13:992799. doi: 10.3389/fpls.2022.992799 (PMC9644038; doi:10.3389/fpls.2022.992799)

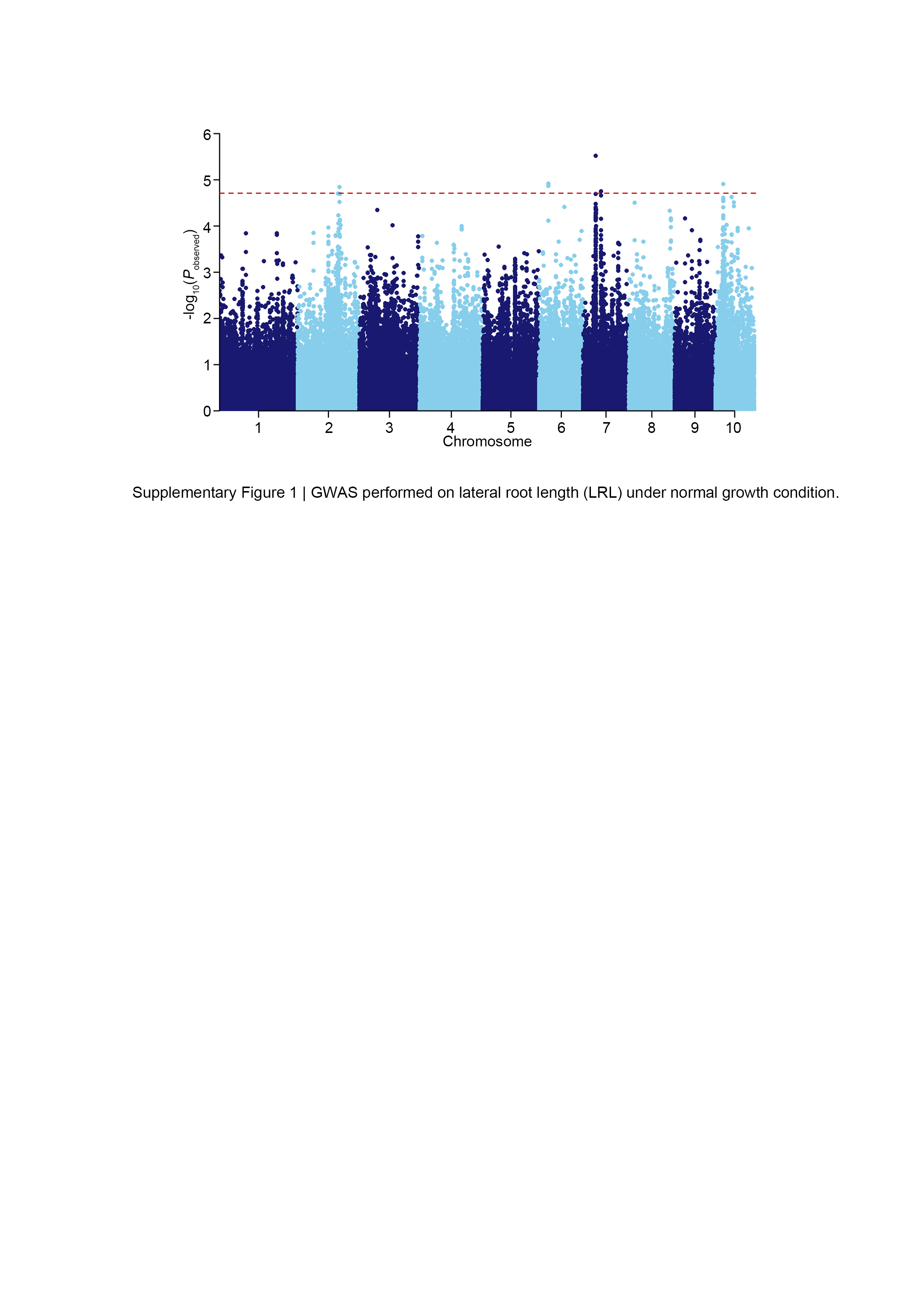

Supplement: Supplementary Figure 1 — GWAS performed on lateral root length (LRL) under normal growth condition. The red horizontal dashed line depicted the significance threshold (P = 1.96 × 10^−5^). [file Image_1.jpeg]

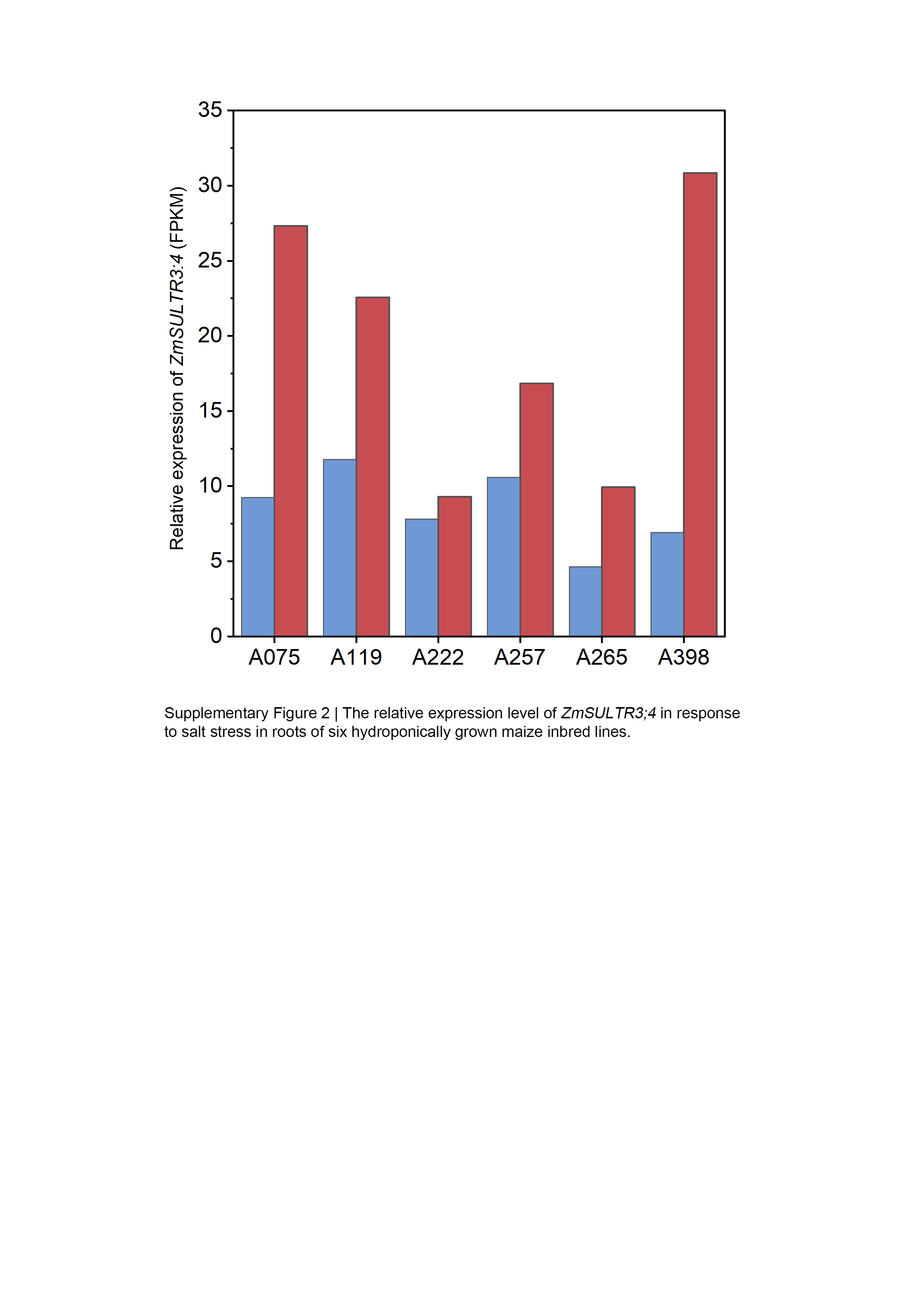

Supplement: Supplementary Figure 2 — The relative expression level of ZmSULTR3;4 in response to salt stress in roots of six hydroponically grown maize inbred lines. The data represented the mean of two replicates. [file Image_2.jpeg]

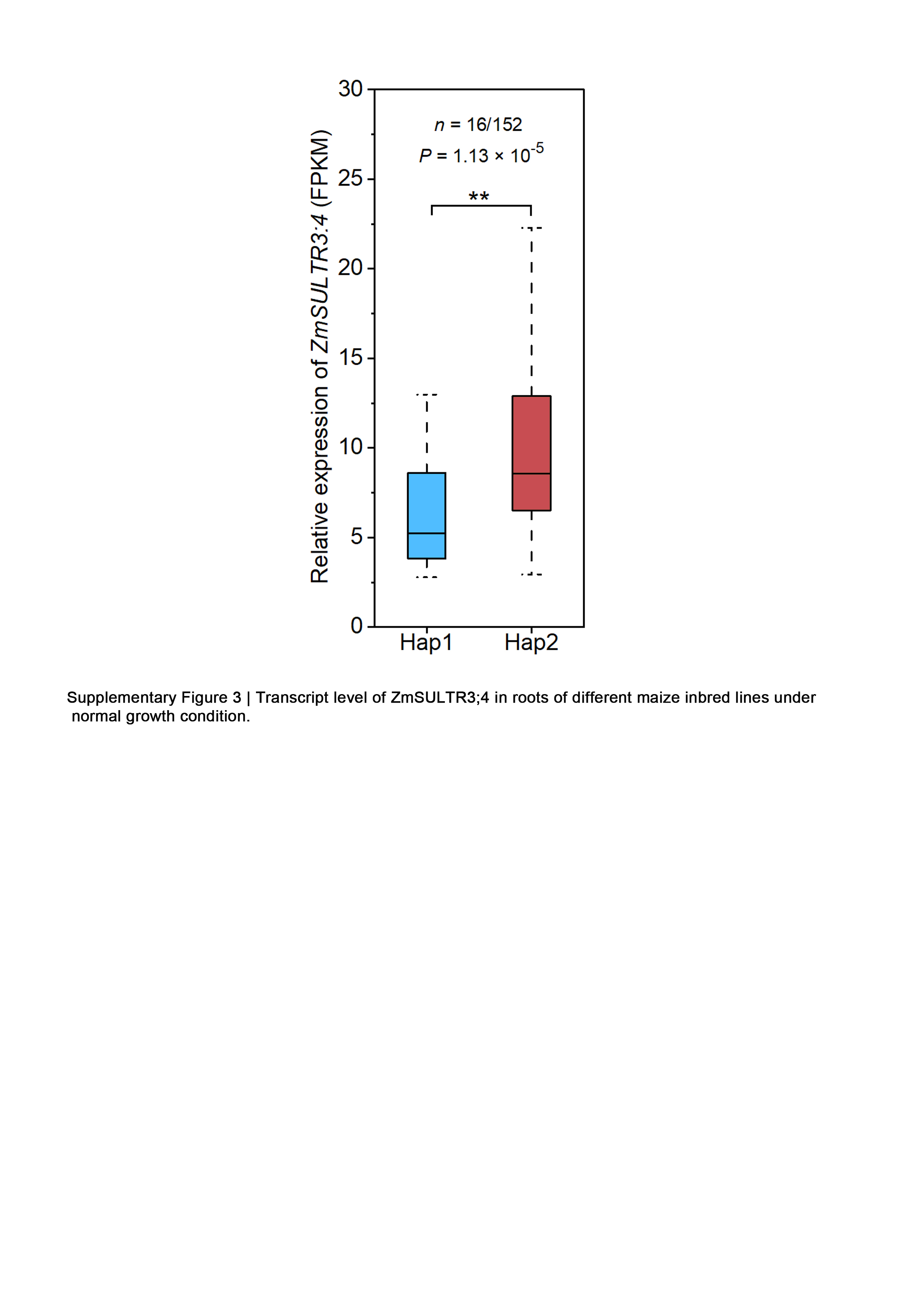

Supplement: Supplementary Figure 3 — Transcript level of ZmSULTR3;4 in roots of different maize inbred lines under normal growth condition. Statistical significance was detected by a two-sided t-test; **p < 0.01. [file Image_3.jpeg]

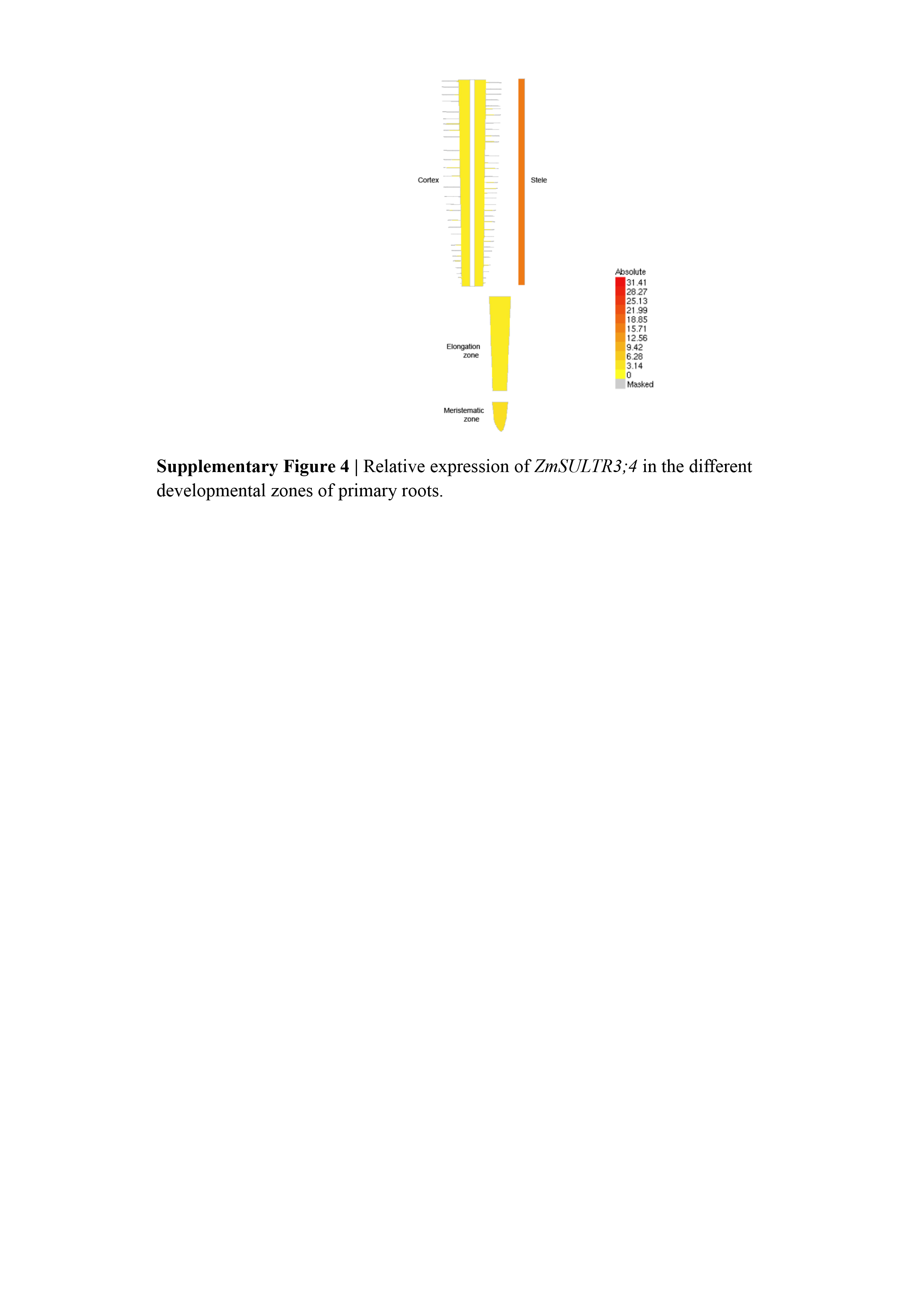

Supplement: Supplementary Figure 4 — Relative expression of ZmSULTR3;4 in the different developmental zones of primary roots. The data were retrieved from the Maize eFP Browser (http://bar.utoronto.ca/efp_maize/cgi-bin/efpWeb.cgi). [file Image_4.jpeg]
